# Chautauqua County Notes

**Published:** 1896-12

**Authors:** 


### Chautauqua County Notes.

The medical society of the county of Chautauqua will hold its next semi-annual meeting at Fredonia, on Tuesday, January 12, 1897. It is proposed to make appendicitis the principal theme for discussion.

Dr. M. N. Bemus, of Jamestown, who has lately spent some time in New York among the various hospitals, has returned and resumed his professional work.

The village of Ripley has been visited with typhoid fever, which has created alarm among the inhabitants as well as a demand for nurses from Buffalo.

Dr. Franklin A. Stevens, late of Sinclairville, and formerly of Dunkirk and Falconer, has located himself in Iowa for the practice of his profession.

Dr. W. A. Putnam, of Westfield, is recovering from a prolonged attack of pneumonia. We trust he will be soon restored to his usual vigor.

Dr. and Mrs. N. G. Richmond, of Fredonia, have lately returned from a fortnight's visit at New York and Boston.

